# Robust Industrial Surface Defect Detection Using Statistical Feature Extraction and Capsule Network Architectures

**DOI:** 10.3390/s25196063

**Published:** 2025-10-02

**Authors:** Azeddine Mjahad, Alfredo Rosado-Muñoz

**Affiliations:** GDDP, Department Electronic Engineering, School of Engineering, University of Valencia, 46100 Burjassot, Valencia, Spain

**Keywords:** defect detection, capsule networks, CNN3D, ResNet50, feature selection, automated visual inspection, casting manufacturing, Industry 4.0

## Abstract

Automated quality control is critical in modern manufacturing, especially for metallic cast components, where fast and accurate surface defect detection is required. This study evaluates classical Machine Learning (ML) algorithms using extracted statistical parameters and deep learning (DL) architectures including ResNet50, Capsule Networks, and a 3D Convolutional Neural Network (CNN3D) using 3D image inputs. Using the Dataset Original, ML models with the selected parameters achieved high performance: RF reached 99.4 ± 0.2% precision and 99.4 ± 0.2% sensitivity, GB 96.0 ± 0.2% precision and 96.0 ± 0.2% sensitivity. ResNet50 trained with extracted parameters reached 98.0 ± 1.5% accuracy and 98.2 ± 1.7% F1-score. Capsule-based architectures achieved the best results, with ConvCapsuleLayer reaching 98.7 ± 0.2% accuracy and 100.0 ± 0.0% precision for the normal class, and 98.9 ± 0.2% F1-score for the affected class. CNN3D applied on 3D image inputs reached 88.61 ± 1.01% accuracy and 90.14 ± 0.95% F1-score. Using the Dataset Expanded with ML and PCA-selected features, Random Forest achieved 99.4 ± 0.2% precision and 99.4 ± 0.2% sensitivity, K-Nearest Neighbors 99.2 ± 0.0% precision and 99.2 ± 0.0% sensitivity, and SVM 99.2 ± 0.0% precision and 99.2 ± 0.0% sensitivity, demonstrating consistent high performance. All models were evaluated using repeated train-test splits to calculate averages of standard metrics (accuracy, precision, recall, F1-score), and processing times were measured, showing very low per-image execution times (as low as $3.69\times 10^{-4}$ s/image), supporting potential real-time industrial application. These results indicate that combining statistical descriptors with ML and DL architectures provides a robust and scalable solution for automated, non-destructive surface defect detection, with high accuracy and reliability across both the original and expanded datasets.

## 1. Introduction

In the automation of industrial quality control, image processing plays a key role in detecting surface defects in components and products. Traditionally, basic processing methods have relied on techniques such as filtering, segmentation, and manual feature extraction. For example, classical filters such as Sobel, Canny, or thresholding techniques have been widely used to highlight edges and defects [1,2]. However, these approaches often require careful calibration and do not adapt well to the inherent variability in industrial images, caused by changes in lighting, noise, or variations in defect type [3,4].

To overcome these limitations, ML techniques have been incorporated to classify defects from features extracted either manually or automatically. Algorithms such as SVM, RF, and KNN have shown good performance in specific visual inspection tasks [5,6]. Nevertheless, these methods are highly dependent on the quality and relevance of the extracted features, and their performance can be affected by issues such as class imbalance or the scarcity of labeled data [7].

In recent years, DL, particularly through convolutional neural networks (CNNs), has revolutionized automatic defect detection in industrial images. CNNs are capable of learning hierarchical representations directly from raw data, eliminating the need for manual feature extraction [8,9]. This has led to significant improvements in detection accuracy and speed across various industrial sectors, including metal manufacturing, textiles, and electronics [10,11].

Despite these advances, the deployment of DL models in industrial environments presents important challenges. Execution times can be high, limiting their integration into real-time production systems. Additionally, these architectures require large amounts of labeled data to achieve robust models, which restricts their applicability in scenarios where defects are rare or evolve over time [11,12]. On the other hand, advanced architectures such as Capsule Networks have shown strong potential for capturing spatial relationships and complex patterns, thereby improving generalization capabilities [8,13].

To address these challenges, hybrid approaches combining statistical feature extraction techniques with ML models and Capsule Network architectures have been explored. These strategies aim to balance accuracy, efficiency, and adaptability, enabling automated visual inspection even under adverse conditions such as class imbalance, data scarcity, and real-time processing requirements [14,15].

In this context, this study presents the design and validation of a hybrid approach for industrial visual inspection, assessing its performance with real data and under scenarios that reflect the conditions and challenges characteristic of the manufacturing industry.

### 1.1. Objective of the Work

This work aims to develop an automated system for defect detection on industrial surfaces through the use of statistical descriptors and artificial intelligence models. Both classical ML algorithms (Random Forest (RF), KNN, Logistic Regression (LR), Gradient Boosting (GB)) and advanced capsule network architectures (Capsule3D, AttnCaps, SpectralCaps, and GraphCaps) are addressed, with the goal of comparing their performance and robustness in real industrial scenarios.

The proposed approach seeks to offer a precise, efficient, and scalable solution capable of adapting to complex conditions such as class imbalance or limited data availability, thereby contributing to the development of intelligent quality control systems aligned with Industry 4.0 principles.

### 1.2. Main Contributions

Based on the proposed approach, this work makes several relevant contributions to the field of automated industrial visual inspection:

- A hybrid framework for detecting industrial surface defects is proposed, combining statistical feature extraction with classical ML models and advanced capsule network architectures.
- A comparative evaluation of five widely used classifiers(RF, KNN, LR, GB, and SVM) against four capsule network variants (Capsule3D, AttnCaps, SpectralCaps, and GraphCaps) is conducted.
- Feature extraction and selection techniques are applied to identify the most relevant parameters for model input and enhance the robustness of the classification process.
- Experimental results show that the combination of statistical descriptors with capsule networks can match or outperform traditional ML models, even in scenarios with limited or imbalanced data.
- A scalable and reliable methodology applicable to real industrial environments is presented, contributing to the advancement of intelligent quality control systems.

The remainder of this article is organized as follows: Section 2 presents the ML and DL models used, along with their theoretical foundations. Section 3 describes the dataset, preprocessing procedures, and employed methodology. Section 3.6 details the training strategy. Section 4, Section 5 and Section 6 present the obtained results, comparative analysis, and the study’s conclusions.

## 2. Foundations of ML and DL

This section presents the basic definitions and models of ML and DL used in this work, along with their theoretical foundations.

### 2.1. ML

ML is a subfield of artificial intelligence focused on developing algorithms capable of learning patterns from data without being explicitly programmed for a specific task [16]. ML algorithms are generally classified into supervised, unsupervised, and semi-supervised learning, with supervised learning being the most commonly used for classification and defect detection problems in industrial contexts.

The ML models used in this work are:

- RF: An ensemble of decision trees trained on random subsets of the data and features. Each tree produces a prediction $h_{t}\left(x\right)$ for an input sample *x*, and the final output is obtained by majority voting:
  (1) $$\hat{y}=\mathrm{mode}\left\{h_{1}\left(x\right),h_{2}\left(x\right),...,h_{T}\left(x\right)\right\}$$
  where *T* is the number of trees in the forest [17].
- SVM: Seeks a hyperplane defined by w and *b* that maximizes the margin between classes. The optimization problem is:
  (2) $$\min_{w,b}\frac{1}{2}{∥w∥}^{2}+C\sum_{i=1}^{n}\xi_{i}$$
  subject to:
  (3) $$y_{i}\left(w^{⊤}\phi \left(x_{i}\right)+b\right)\geq 1-\xi_{i},\;\xi_{i}\geq 0$$
  where ϕ(·) is the feature mapping function, $\xi_{i}$ are slack variables, and *C* is a regularization parameter [18].
- LR: Models the probability of belonging to the positive class as:
  (4) $$P\left(y=1|x\right)=\frac{1}{1+e^{-(w^{⊤}x+b)}}$$
  and is trained by maximizing the log-likelihood function [19]:
  (5) $$L\left(w,b\right)=\sum_{i=1}^{n}y_{i}\log P\left(y_{i}|x_{i}\right)+\left(1-y_{i}\right)\log\left(1-P\left(y_{i}|x_{i}\right)\right)$$
- KNN: Classifies a sample x based on the majority label among its *k* closest neighbors, according to a distance metric $d(x,x_{i})$, typically Euclidean:
  (6) $$d\left(x,x_{i}\right)=\sqrt{\sum_{j=1}^{m}{\left(x_{j}-x_{ij}\right)}^{2}}$$
  where *m* is the number of features [20].
- GB: Builds an additive model:
  (7) $$F_{m}\left(x\right)=F_{m-1}\left(x\right)+\nu h_{m}\left(x\right)$$
  where $h_{m}$ is a weak learner fitted to the negative gradient of the loss function *L* with respect to the predictions $F_{m-1}$, and ν is the learning rate [21].

### 2.2. DL

DL involves the use of neural networks with multiple layers to model complex and abstract patterns in data. Traditional convolutional neural networks CNNs) have achieved great success in many image-related tasks; however, they often struggle to preserve detailed spatial hierarchies and pose information.

Capsule networks represent an advancement in DL architectures designed to better capture and preserve spatial relationships and hierarchical features within the data [22]. Unlike CNNs, capsules output vectors that encode both the presence and pose of features, improving robustness to spatial transformations.

**Figure 1:**
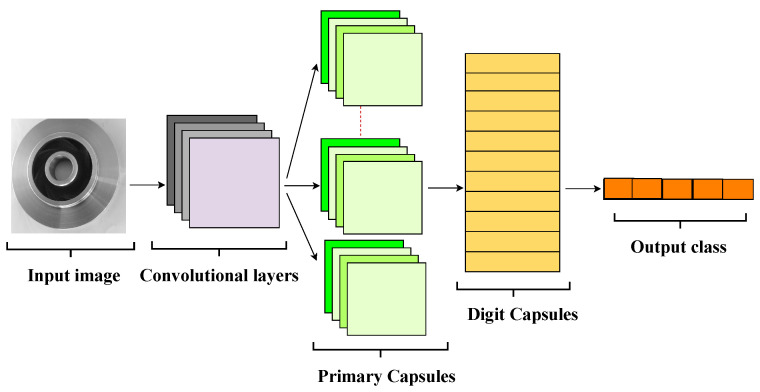
General architecture of a Capsule Network, showing the key layers and operations.

Figure 1 illustrates the general architecture of a capsule network, highlighting its main components:

The general architecture of a capsule network includes the following main components:

- Input Layer: Receives raw or preprocessed image data.
- Convolutional Layer: Extracts low-level features such as edges and textures, similar to CNNs.
- Primary Capsules: Groups of neurons organized into capsules that encode simple features as vectors representing their presence and pose (orientation, position, scale).
- Digit Capsules: Higher-level capsules that receive input from primary capsules and encode more complex features representing specific classes (e.g., defect categories).
- Squash Function: A specialized activation function applied to normalize the length of capsule output vectors between 0 and 1, preserving pose information. The function is defined as:
  (8) $$v_{j}=\frac{\left|\right|s_{j}{\left|\right|}^{2}}{1+\left|\right|s_{j}{\left|\right|}^{2}}\frac{s_{j}}{\left|\right|s_{j}\left|\right|}$$
  where $s_{j}$ is the input vector to capsule *j* and $v_{j}$ is the normalized output vector.
- Decision Layer: Computes the length (norm) of each class capsule’s output vector, interpreting it as the probability of the corresponding class, with the final prediction corresponding to the capsule with the greatest length.

#### Advanced Capsule Network Variants

In this work, several advanced capsule network variants are employed, tailored for industrial defect detection:

- Capsule3D: Extends the capsule concept to three-dimensional data, where each capsule is a vector $v\in R^{d}$ representing instantiation parameters. The transformation between capsules is done through weight matrices $W_{ij}$:
  (9) $${\hat{u}}_{j|i}=W_{ij}u_{i}$$
  where $u_{i}$ is the output of capsule *i* in the lower layer, and ${\hat{u}}_{j|i}$ is the prediction for capsule *j* in the upper layer [23].
- AttnCaps: Introduces an attention mechanism assigning weights $\alpha_{ij}$ to connections between capsules to emphasize the most relevant relationships. The output of an upper-level capsule is computed as:
  (10) $$s_{j}=\sum_{i}\alpha_{ij}{\hat{u}}_{j|i},\;\alpha_{ij}=\frac{\exp(e_{ij})}{\sum_{k}\exp\left(e_{kj}\right)}$$
  where $e_{ij}$ is the compatibility score between capsules [24].
- SpectralCaps: Applies spectral transformations (e.g., Fourier or Wavelet transforms) to capsule outputs to capture frequency-domain information:
  (11) $$v^{\left(f\right)}=F\left(v\right)$$
  where F denotes the spectral transform, enhancing texture and pattern detection [25].
- GraphCaps: Combines capsule networks with graph structures to model non-Euclidean relationships among capsules. Given a graph G=(V,E) with nodes *V* representing capsules and edges *E* their connections, the capsule states update as:
  (12) $$h_{v}^{\left(l+1\right)}=\sigma \left(\sum_{u\in N(v)}W^{\left(l\right)}h_{u}^{\left(l\right)}+b^{\left(l\right)}\right)$$
  where $h_{v}^{\left(l\right)}$ denotes the state of capsule *v* at layer *l*, N(v) represents its neighbors, and σ is an activation function [26].

This theoretical framework provides a solid foundation for comparing classical CNNs and advanced capsule-based methods in industrial surface defect detection.

## 3. Materials and Methodology

This study proposes a hybrid approach for detecting defects on industrial surfaces, combining statistical descriptors with ML algorithms and advanced DL architectures, specifically Capsule Networks.

**Figure 2:**
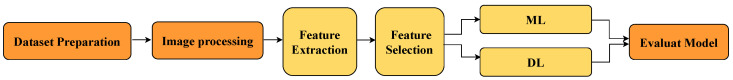
Flowchart of the proposed system’s training process.

The methodology is divided into two main phases: training and evaluation, as illustrated in the flowchart shown in Figure 2. Each phase consists of multiple stages briefly described below.

### 3.1. Training Phase

#### 3.1.1. Dataset Preparation

Labeled images of industrial products were used, categorized as defective or non-defective. The dataset was split into training and test subsets using stratified sampling to preserve the original class proportions. All images were processed in RGB color space. No data augmentation was applied in this phase.

#### 3.1.2. Preprocessing

Images were resized to a uniform size and intensity-normalized to ensure compatibility with all models.

#### 3.1.3. Feature Extraction

Previously extracted and selected parameters were used as input for both ML and DL models. Additionally, for a third case, full images were converted into a simulated hyperspectral cube, which was used exclusively as input for the 3D CNN.

#### 3.1.4. Model Training and Optimization

The ML classifiers used were: RF, SVM, LR, KNN, and GB. Each model was trained using a validation subset held out from the training set for hyperparameter tuning. Five repetitions of random subsampling were performed to provide stable performance estimates. Capsule networks were trained via backpropagation using loss functions adapted for binary classification, following the same division and validation scheme.

### 3.2. Evaluation Phase

**Figure 3:**
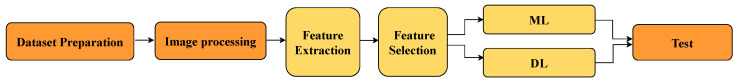
Flowchart of the evaluation phase of the proposed system.

As illustrated in Figure 3, the evaluation phase follows the workflow for testing and performance measurement of all models.

#### 3.2.1. Test Set Preprocessing

Test images underwent the same preprocessing steps applied during training to ensure consistent input.

#### 3.2.2. Model Evaluation

The same inputs were used in all three cases: previously extracted and selected parameters, full images, and simulated hyperspectral cubes (only for the 3D CNN). Both ML and DL models were evaluated in all cases.

#### 3.2.3. Performance Measurement

Model performance was assessed using standard classification metrics: accuracy, recall (sensitivity), precision, F1-score, area under the ROC curve (ROC-AUC), and area under the Precision-Recall curve (PR-AUC). Confusion matrices and ROC/PR curves were used to evaluate the models’ discriminative ability.

### 3.3. Dataset

The dataset used in this study comes from Cast Components with Surface Defects, originally available on Kaggle. It contains images of industrial cast parts annotated based on the presence or absence of surface defects. After carefully checking the data sources and previous works that have used this dataset, we note that none of the prior studies mention the name or model of the camera used. The images were captured using a camera sensor under stable lighting conditions.

Two versions of the dataset are available:

- Dataset Original → 1300 grayscale images of 512 × 512 pixels without augmentation, divided into two classes: normal (parts without visible surface anomalies, OK_FRONT) and defective (parts with flaws such as porosity, open holes, flashing, cracks, and stains, DEF_FRONT).
- Dataset Expanded → 7348 grayscale images of 300 × 300 pixels with augmentation applied, organized into training (train) and testing (test) folders, also divided into the same two classes.

For this work, only the Dataset Original was used. All images were converted to 3 color channels (RGB) and normalized to the [0, 1] range. The analysis was performed using a cross-validation scheme, without additional data augmentation.

The defective class is treated as the positive class for all experiments, and predictions are binarized using a decision threshold of 0.5 to compute metrics such as accuracy, precision, recall, and F1-score.

**Figure 4:**
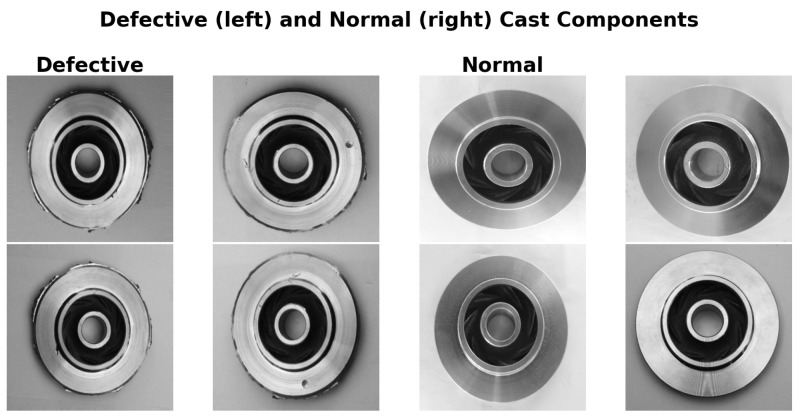
Representative cropped regions from defective samples in the dataset, highlighting structural cracks, blow holes, flashing, and stains.

Representative cropped regions of defective samples are shown in Figure 4, highlighting structural cracks, blow holes, flashing, and stains. Although the Dataset Expanded allows direct model training and evaluation, it was not used in this study.

The defective samples include various types of anomalies, reflecting the diversity of defects in the industrial domain considered.

### 3.4. Feature Extraction and Selection

For each image in the dataset, a comprehensive set of descriptors was calculated, covering multiple domains, including statistical, texture, shape, frequency, entropy, and complexity, in both spatial and transformed domains (frequency and multiscale). The initial set contained approximately 130 parameters, a number chosen to capture the maximum information present in the images. Many descriptors include multiple variants or are summarized through statistics, considering different distances, angles, or regions, ensuring a rich representation of the patterns in the data before any selection process.

To reduce redundancy and improve computational efficiency, a correlation-based feature selection method was applied. Absolute correlations between each feature and the target class were calculated, and highly correlated variables among themselves were removed based on a predefined threshold.

Finally, Principal Component Analysis (PCA) was applied on the selected features to assess their relative importance in explaining the total variance. PCA loadings provide a measure of each feature’s contribution, guiding the identification of the most relevant parameters for subsequent modeling.

Table 1 presents a representative subset of these descriptors, organized by feature domain, along with their descriptions.

### 3.5. 3D Convolutional Neural Network Approach with Simulated HSI

In addition to the feature-based ML/DL approaches, we implemented a CNN3D. The key idea was to exploit both spatial and spectral information by converting each RGB image into a simulated hyperspectral cube.

#### 3.5.1. HSI Simulation

Each RGB image was resized to 128×128 pixels and normalized to [0,1]. The grayscale version was then replicated across 10 spectral bands, resulting in a 3D input of shape 128×128×10 for the CNN. This allowed the network to learn features along both spatial (height and width) and spectral (bands) dimensions.

#### 3.5.2. Visualizations of Simulated HSI

**Figure 5:**
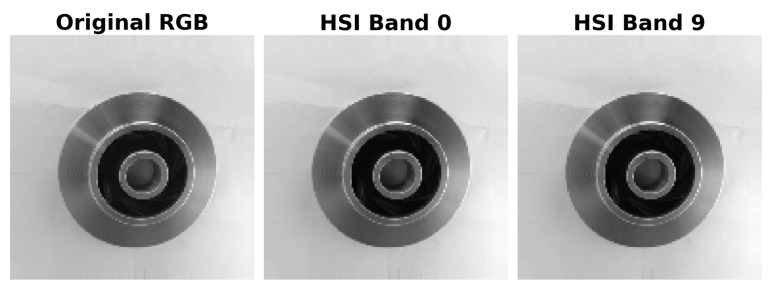
Comparison of original RGB image and individual bands of the simulated HSI cube.

**Figure 6:**
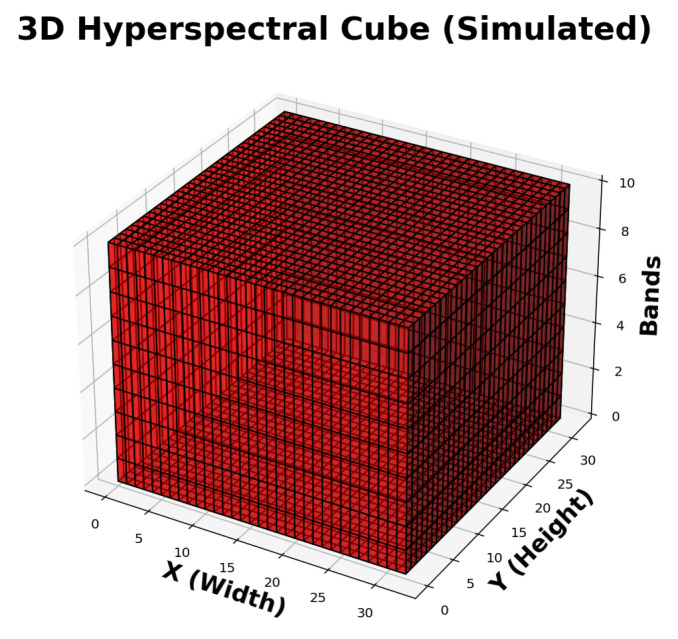
3D visualization of the simulated hyperspectral cube. Voxels indicate intensity across spatial and spectral dimensions.

Figure 5 shows the original RGB image alongside two bands of the simulated hyperspectral cube, while Figure 6 presents a 3D representation highlighting its spatial–spectral structure.

### 3.6. Model Training and Evaluation

For all approaches—ML models, DL Capsule Networks, and the CNN3D—the same data splitting strategy was applied: 80% of the dataset for training and 20% for testing. Within the training set, 20% was held out for validation and hyperparameter tuning. To ensure robustness, repeated random subsampling validation with 5 repetitions was performed using fixed random seeds, and performance metrics were averaged across runs.

#### 3.6.1. CNN3D Architecture

The network consisted of two 3D convolutional layers with ReLU activations, each followed by 3D max-pooling layers. The resulting feature maps were flattened and passed through two fully connected layers, ending in a softmax output layer. Training used the Adam optimizer and categorical crossentropy loss, with a batch size of 8 for 10 epochs.

As shown in Table 2, this architecture demonstrates how the CNN3D extracts hierarchical features from simulated hyperspectral inputs, complementing feature-based ML and conventional DL methods.

#### 3.6.2. Evaluation

Performance was assessed using accuracy, precision, recall, F1-score, ROC-AUC, and PR-AUC. For the CNN3D, visualizations of RGB inputs, HSI bands, and 3D cubes were generated to illustrate the input representation. This unified framework ensures that all models were trained and evaluated under the same conditions, enabling a fair performance comparison.

### 3.7. Evaluation Metrics

The performance of the evaluated models—both supervised (ML and DL) and the CNN3D—was measured using standard binary classification metrics: accuracy, recall, precision, F1-score, ROC-AUC, and PR-AUC [27]. For all metrics, the defective class was treated as positive, with predictions binarized at a 0.5 threshold. The ROC curve summarizes the trade-off between true positive rate (TPR) and false positive rate (FPR), while the PR curve highlights the precision–recall relationship, particularly useful under class imbalance.

## 4. Results

This section presents the outcomes obtained from applying various classifiers to the task of detecting surface defects in cast components.

For all experiments presented in this section, the Dataset Original was used, unless otherwise specified. Experiments using the Dataset Expanded are only reported for traditional ML classifiers (RF, LR, KNN, SVM, GB), to evaluate performance on a larger dataset.

To enhance model performance, hyperparameter tuning was performed for all algorithms that support it.

**Figure 7:**
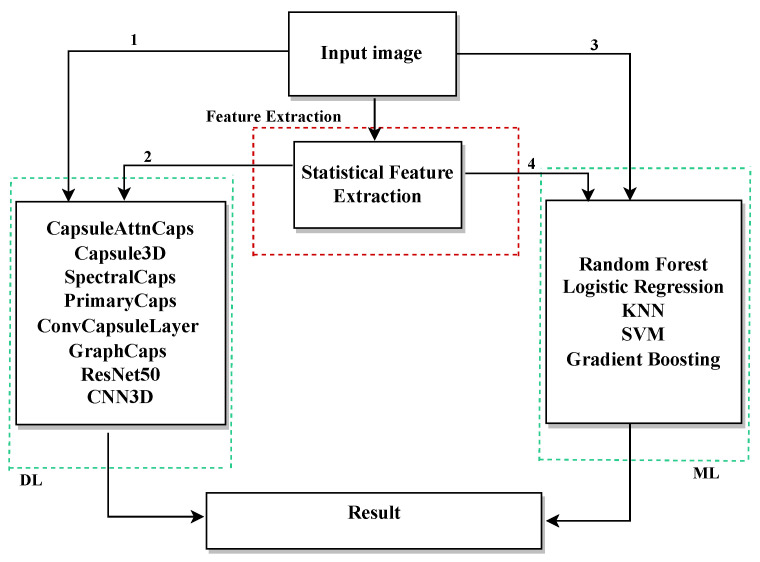
Overview of the proposed methodological pipeline, illustrating four processing paths for defect detection. Numbers 1–4 correspond to the paths described in the Results section.

Before discussing specific results, Figure 7 provides an overview of the proposed methodological pipeline. Starting from preprocessed images, four distinct processing paths were explored:

- Path 1: Raw images are directly input into DL models such as AttnCapsNet, 3D Capsule3D, SpectralCap, PrimaryCaps, ConvCaps, GraphCaps, and ResNet50.
- Path 2: DL models are employed to extract abstract features from images, which are subsequently used for classification within DL architectures.
- Path 3: Raw images are directly used as input to traditional ML classifiers, including RF, LR, KNN, SVM, and GB.
- Path 4: Statistical features are extracted from images and then used as input for the same set of ML classifiers.

In addition, the CNN3D with simulated HSI approach (described in Section 3.5) was also evaluated, where RGB images were converted into hyperspectral-like cubes to exploit spatial–spectral information.

### 4.1. Feature Extraction and Correlation-Based Selection

All analyses in this study are based on the Dataset Original, consisting of 1300 images. From the raw images, an initial set of 134 statistical parameters was extracted, along with one target variable (Class), resulting in a dataset of 1300 samples by 135 columns.

To improve model efficiency and remove redundancy, a correlation-based feature selection method was applied. First, the absolute correlation between each parameter and the target class was calculated. Then, highly correlated features (correlation >0.9) were removed, yielding a final set of 26 key parameters.

**Figure 8:**
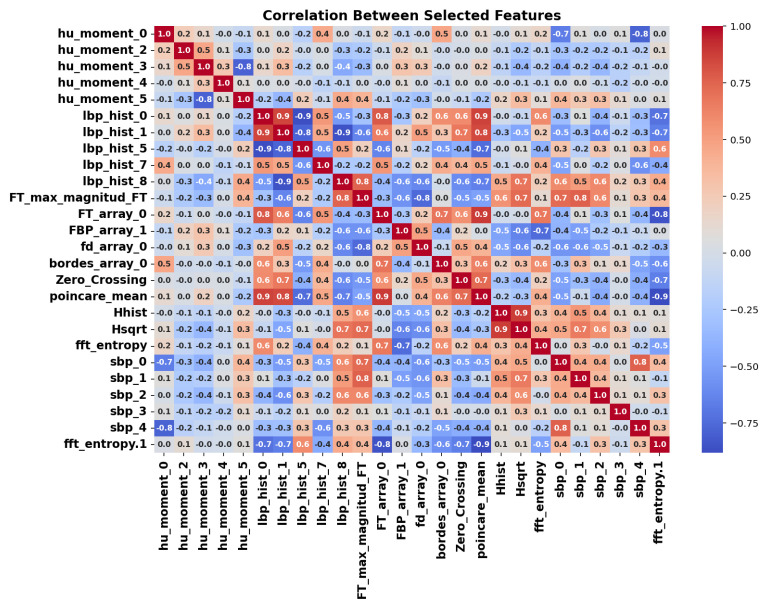
Correlation matrix of the 26 selected features after applying correlation-based dimensionality reduction.

Figure 8 shows the correlation matrix of the selected features, confirming that the remaining parameters are sufficiently decorrelated and have low redundancy. This preserves complementary information, reduces dimensionality, and improves the robustness of subsequent ML and DL models.

### 4.2. Statistical Significance Evaluation

Univariate statistical tests (ANOVA or Mann-Whitney, depending on the distribution of each variable) were applied to assess the discriminative power of the parameters. False Discovery Rate (FDR) correction was used to account for multiple comparisons.

**Figure 9:**
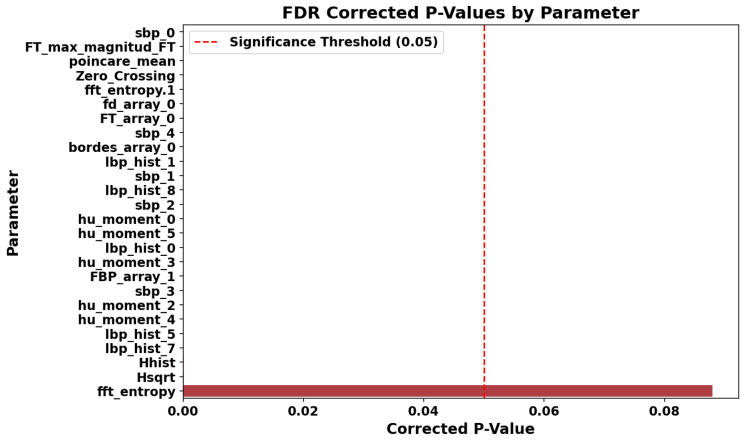
Adjusted *p*-values from univariate statistical tests with FDR correction for the selected features. The red shaded area indicates the significance threshold (p<0.05), and parameters with *p*-values within this area are considered statistically significant.

The results showed that 25 out of the 26 selected parameters exhibited statistically significant differences between classes (p<0.05), as summarized in Figure 9. The parameter fft_entropy was the only one not reaching significance but was retained due to its relevance in multivariate analysis.

### 4.3. Feature Importance Analysis via PCA

PCA was applied to the 26 selected features to assess their relative importance in explaining total variance. The absolute values of the loadings on the first five principal components were summed for each variable.

The top 10 most important parameters identified by the PCA analysis are presented in Table 3. These parameters represent a rich combination of structural, frequency, and texture descriptors.

### 4.4. Exploratory Visual Analysis

**Figure 10:**
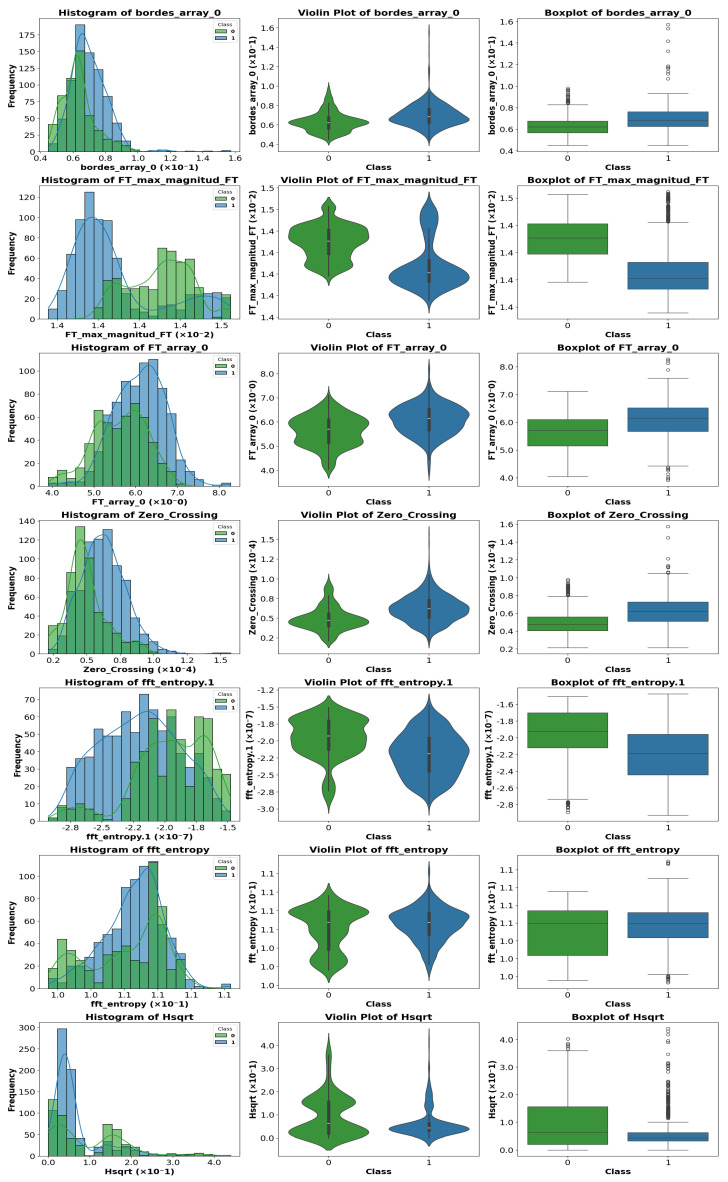
Representative example of violin plot, boxplot, and histogram visualization for selected features. In the boxplots, the horizontal line indicates the median, the box shows the interquartile range, and the whiskers extend to 1.5 × IQR. White circles represent outliers, while blue and green vertical lines indicate class-specific reference values.

**Figure 11:**
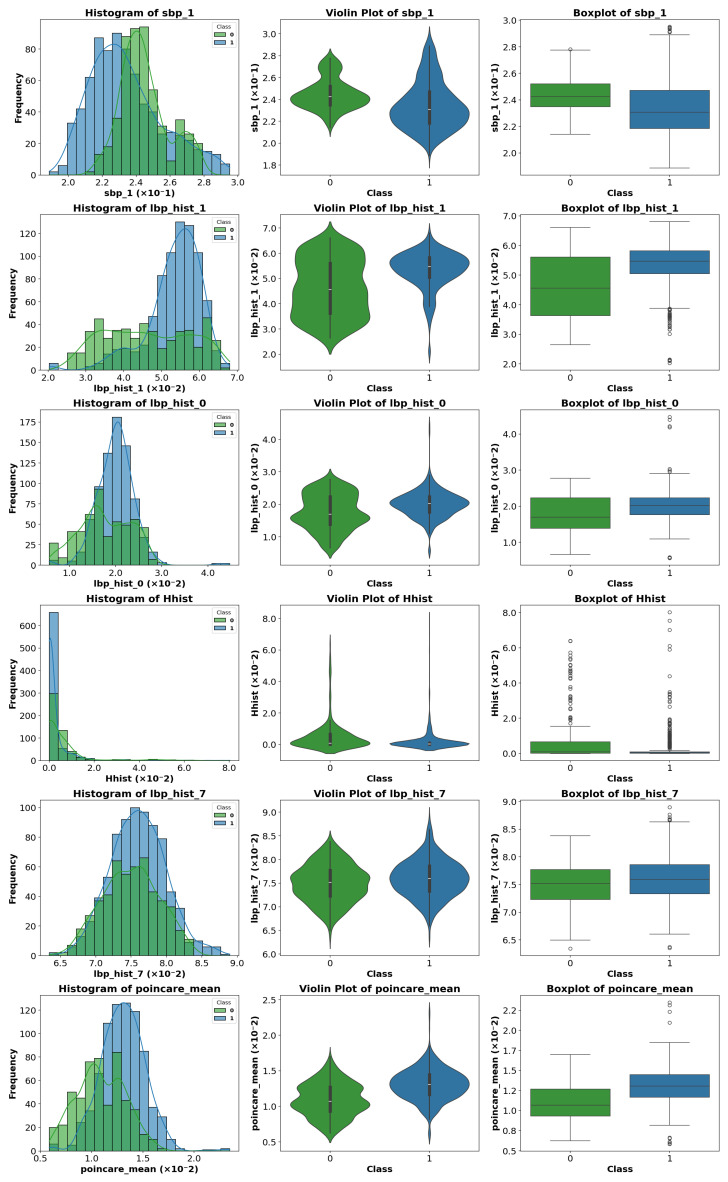
Representative example of violin plot, boxplot, and histogram visualization for selected features. In the boxplots, the horizontal line indicates the median, the box shows the interquartile range, and the whiskers extend to 1.5 × IQR. White circles represent outliers, while blue and green vertical lines indicate class-specific reference values.

**Figure 12:**
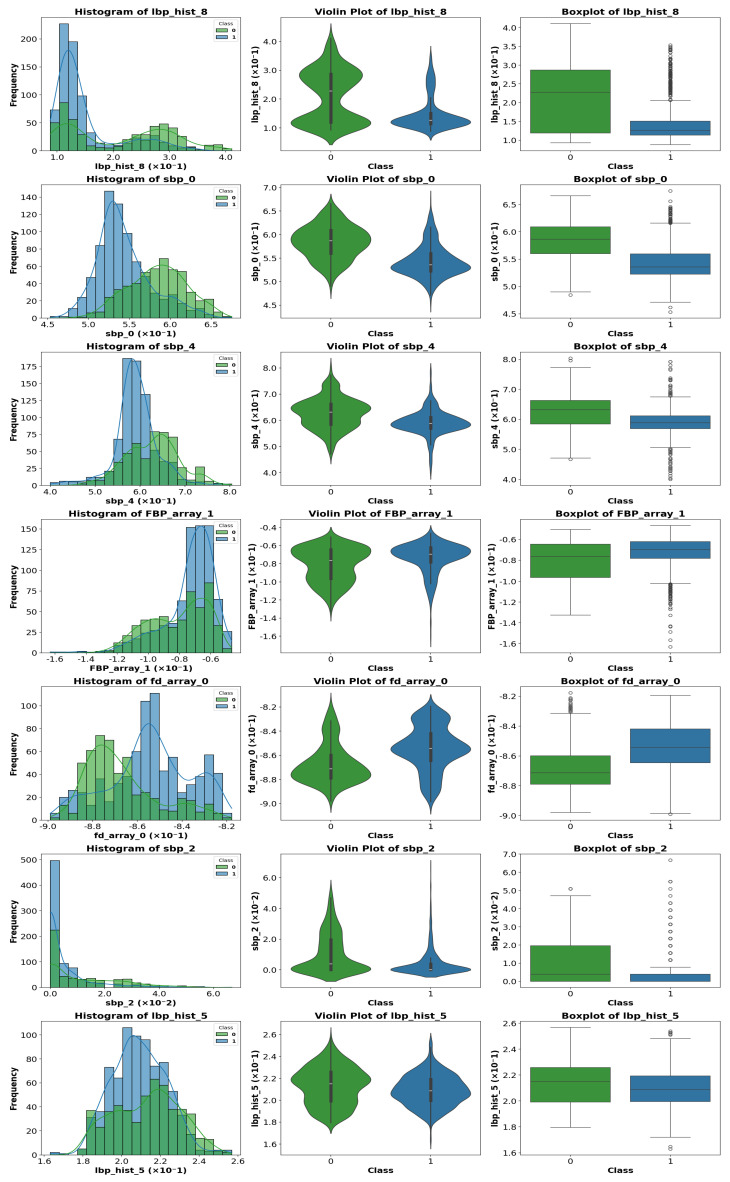
Representative example of violin plot, boxplot, and histogram visualization for selected features. In the boxplots, the horizontal line indicates the median, the box shows the interquartile range, and the whiskers extend to 1.5 × IQR. White circles represent outliers, while blue and green vertical lines indicate class-specific reference values.

To visualize differences between classes for the most relevant parameters, various graphical representations were generated, including histograms, violin plots, and boxplots. Figure 10, Figure 11 and Figure 12 show representative examples of these analyses.

Clear differences were observed in medians, dispersion, and the presence of outliers, confirming the usefulness of these features for the classification task. These visualizations provide an intuitive understanding of how certain features vary across different classes.

A visual inspection of the plots revealed that some parameters exhibit stronger discriminative power than others. Therefore, instead of analyzing the entire feature set, we focus on those parameters that show the most significant class separability as well as those that perform poorly. This selective analysis offers insights into the strengths and limitations of the extracted features.

### 4.5. Dataset Overview for Modeling

After rigorous filtering and statistical analysis, the final dataset used as input for the ML and DL classifiers comprised:

- Number of samples: 1300
- Number of selected parameters: 10

This refined dataset facilitated more efficient model training and significantly enhanced classifier performance, as will be shown in the subsequent results section.

### 4.6. ML

This section presents the results obtained by applying classical classification models under two different configurations. In the first configuration, the models use raw images as input without any prior feature extraction. In the second configuration, relevant parameters are extracted from the images and used as input features for the classifiers. The goal is to compare how the input type affects model performance.

Table 4 lists the classical ML models used in this study along with their default hyperparameters. These configurations were applied consistently across all experiments to ensure a fair comparison.

Table 5 presents the results of the ML models when raw images are used as input, without any prior feature extraction. It can be observed that tree-based models such as RF andGB achieve the highest accuracy, around 85–89%, while K-Nearest Neighbors performs slightly lower. LR shows the lowest performance, suggesting that linear decision boundaries are insufficient when using raw image input.

Table 6 shows the results obtained when the 10 selected parameters extracted from images are used as input for classical ML models. In this configuration, performance improves noticeably, particularly for RF andGB, which reach accuracies above 94%. Sensitivity and specificity are also higher, and the variance across repetitions (±values) is reduced, indicating more stable and reliable predictions. LR and K-Nearest Neighbors also benefit from feature extraction, although the improvements are less pronounced.

All metrics were calculated using multiple repetitions of training and testing splits, with the final reported results being the average across these repetitions. This approach ensures that the performance evaluation reflects a robust estimate rather than relying on a single train/test split.

Finally, the processing time per image for each model using extracted parameters is reported in Table 7. All models are highly efficient, with processing times in the sub-millisecond range, making the use of extracted parameters suitable for real-time or batch applications.

Overall, these results indicate that feature extraction significantly enhances ML performance, particularly for models capable of leveraging structured input, and provides faster, more consistent predictions compared to using raw images directly.

**Figure 13:**
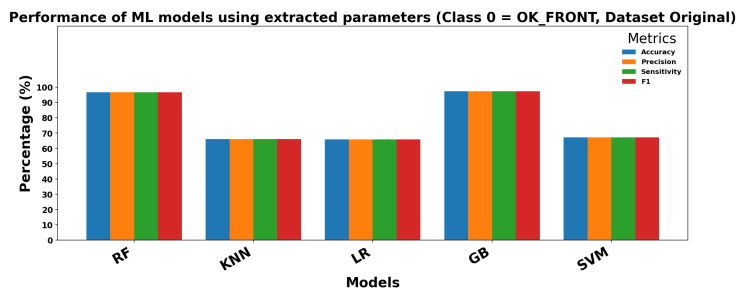
Performance of ML models for Class 0 (OK-FRONT).

**Figure 14:**
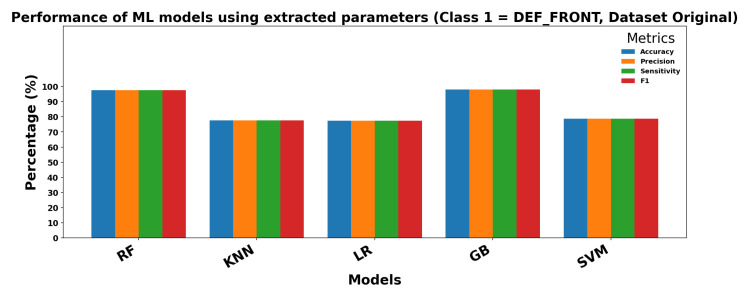
Performance of ML models for Class 1 (DEF-FRONT).

Figure 13 and Figure 14 show the performance of ML models using extracted parameters on the Dataset Original for Class 0 (OK_FRONT) and Class 1 (DEF_FRONT), respectively. Metrics include Accuracy, Precision, Sensitivity, and F1 score, showing high performance across both classes.

### 4.7. DL

This section presents the performance of Capsule-based DLarchitectures. Two configurations are evaluated: (1) using raw images as input and (2) combining the input with extracted parameters.

The results obtained using DL architectures were evaluated under two configurations: using raw images as input and using extracted parameters.

Using raw images, the results are presented in Table 8 and Table 9. ResNet50 shows uneven performance: the normal class is correctly classified in approximately 42–65% of the cases on average, while the affected class reaches 100% in some cases, indicating confusion with the normal class. In contrast, capsule-based models (AttnCapsNet, ConvCaps, 3D CapsNet, Primary, Capsule 3D) achieve sensitivity and precision above 89% for both classes, with F1 scores around 90–93%, demonstrating more consistent and reliable classification. The Spectral model performs worse, particularly for the normal class (76–77% sensitivity), highlighting the need for more discriminative architectures.

When using extracted parameters, the results are shown in Table 10 and Table 11. Incorporating extracted features markedly improves performance. ResNet50 now reaches sensitivities and precisions above 97% for both classes, with F1 scores around 97–98%, demonstrating that feature extraction helps standard architectures learn more discriminative characteristics. Capsule and Graph-based models achieve even higher performance, with sensitivities and precisions exceeding 97% for both classes, F1 scores close to 98–100%, and overall accuracies above 98%, indicating excellent class discrimination. The Spectral model improves when using parameters but remains lower than the capsule and Graph models.

Average processing times per image are reported in Table 12. All models exhibit very low processing times on the order of milliseconds. The Primary model is slightly slower (1.565 $\times \;10^{-3}$ s/image), but this increase is minimal compared to the substantial performance gains from using extracted parameters. Other models process each image in 3.69–9.17 $\times \;10^{-4}$ s, ensuring practical applicability.

CNN3D processes 3D images directly without feature extraction. As shown in Table 13, it achieves high sensitivity for the normal class (91%) and good overall accuracy (88%), demonstrating stable performance, although slightly lower than the best Capsule-based models with extracted features.

Overall, these results show that while raw image input can achieve reasonable performance with complex architectures like capsules, incorporating extracted parameters significantly enhances both standard and capsule-based networks, providing more reliable and discriminative classification.

**Figure 15:**
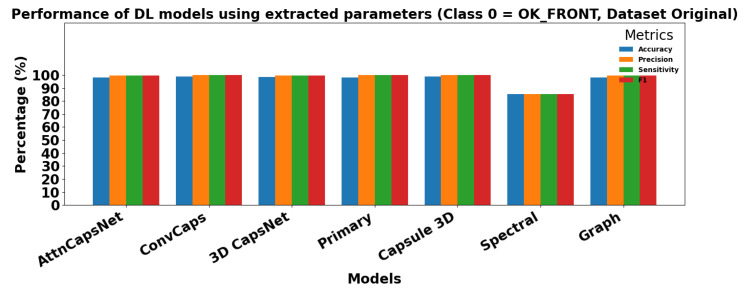
Performance of DL models using extracted parameters for Class 0 (OK-FRONT).

**Figure 16:**
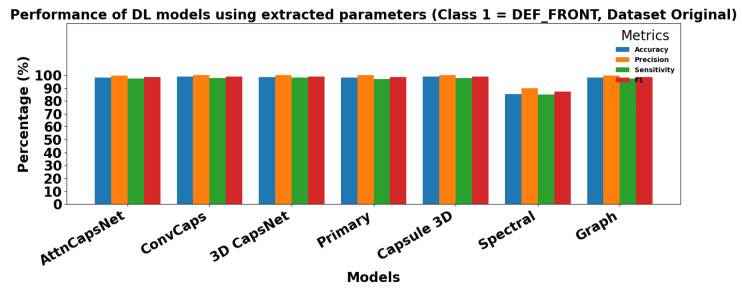
Performance of DL models using extracted parameters for Class 1 (DEF-FRONT).

Figure 15 and Figure 16 show the performance of DL models using extracted parameters on the Dataset Original for Class 0 (OK_FRONT) and Class 1 (DEF_FRONT), respectively. Metrics include Accuracy, Precision, Sensitivity, and F1 score, demonstrating very high performance for both classes.

The results obtained using classical Machine Learning models with extracted parameters (PCA = 100) are presented in Table 14.

Overall, all models achieve excellent performance, reflecting the high discriminative power of the extracted features. RF and SVM show near-perfect classification for both classes, with precision, sensitivity, and F1 scores consistently above 99%. K-Nearest Neighbors also performs exceptionally well, slightly lower for the affected class but still above 98% in all metrics. GB achieves high scores around 96–97%, and LR shows slightly lower performance, particularly for the normal class (precision and sensitivity around 83–84%), while the affected class reaches above 90%.

These results indicate that when using extracted features, classical ML models can classify normal and affected cases almost perfectly, minimizing confusion between classes. The extracted parameters provide sufficient information for the models to distinguish patterns reliably, removing the need for further feature engineering or data extraction procedures.

**Figure 17:**
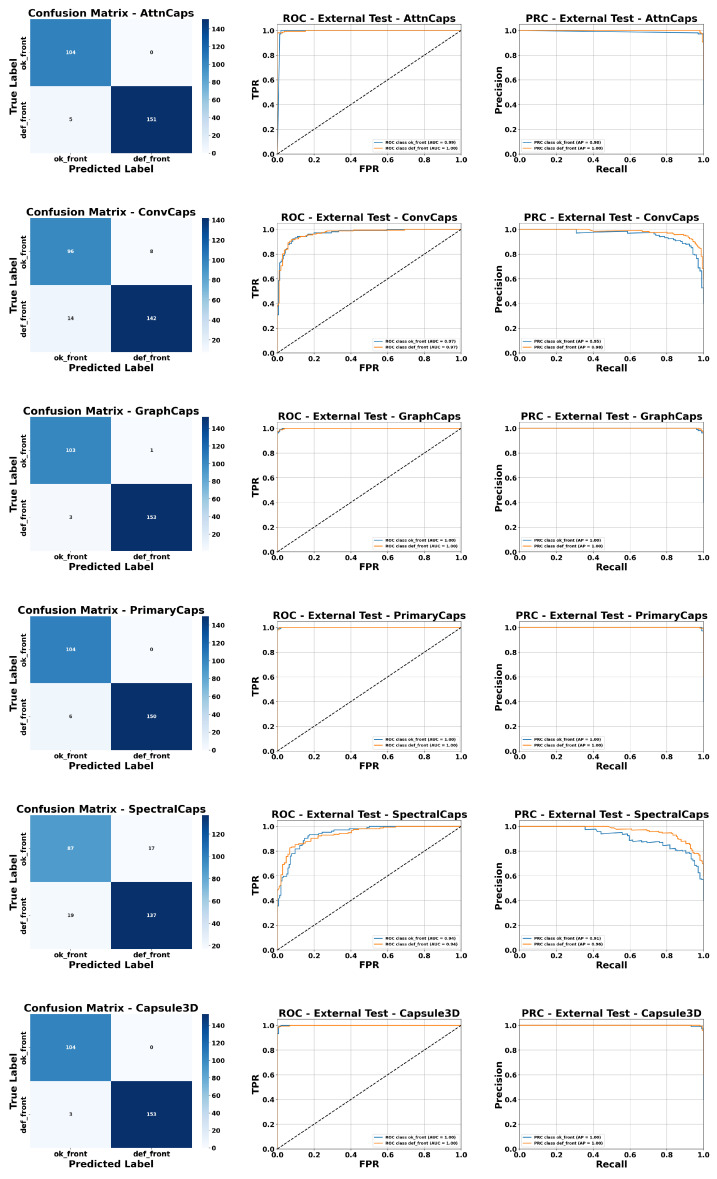
Visual evaluation of model performance using CM, ROC, and PRC curves with combined input (image + parameters). The black dashed line indicates the reference baseline (random classifier) for ROC and PRC curves.

Figure 17 presents a visual evaluation of the DL models’ performance using combined inputs (images + extracted parameters). Confusion matrices (CM), ROC curves, and Precision-Recall (PRC) curves are shown for all evaluated Capsule architectures.

Starting with AttnCaps, the confusion matrix indicates that both classes, DEF_FRONT and OK_FRONT, are correctly classified, reflecting excellent performance. The ROC curve shows AUC values of 0.99 for OK_FRONT and 1.0 for DEF_FRONT. In the PRC, the average precision (AP) is 0.98 for OK_FRONT and 1.0 for DEF_FRONT, confirming the model’s high precision and sensitivity.

For ConvCaps, AUC values are around 0.9 for both classes, while PRC curves show AP = 0.95 for OK_FRONT and 0.98 for DEF_FRONT, indicating solid performance, though slightly lower than AttnCaps.

The GraphCaps and PrimaryCaps models demonstrate outstanding performance, achieving AUC = 1 for both classes and AP = 1 in PRC curves, reflecting perfect classification on this test set.

The SpectralCaps model achieves AUC = 0.94 for both classes, with AP = 0.91 for OK_FRONT and 0.96 for DEF_FRONT, indicating very high performance, albeit slightly lower than the previously mentioned models.

Finally, Capsule3D, like GraphCaps and PrimaryCaps, reaches AUC = 1 and AP = 1 for both classes, demonstrating its ability to exploit the spatial and spectral information in the simulated 3D inputs.

Overall, these visualizations confirm that integrating statistical parameters with Capsule architectures enables highly precise and robust class discrimination, with GraphCaps, PrimaryCaps, and Capsule3D standing out in terms of AUC and AP.

**Figure 18:**
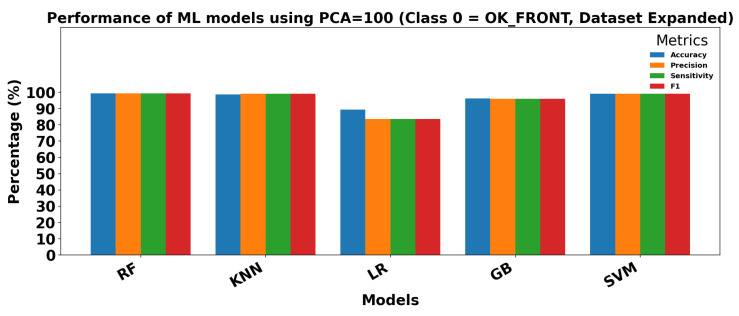
Performance of ML models using extracted parameters (PCA = 100) for Class 0 (OK-FRONT).

**Figure 19:**
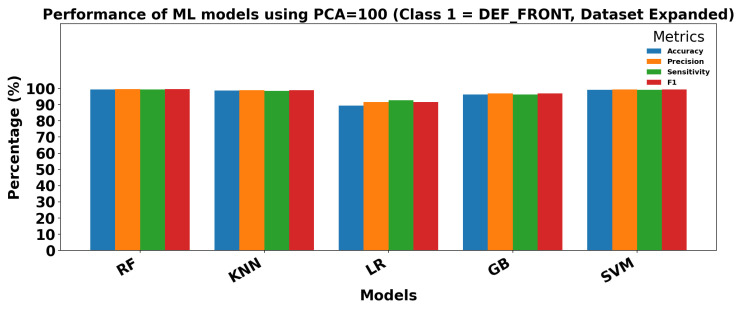
Performance of ML models using extracted parameters (PCA = 100) for Class 1 (DEF-FRONT).

Figure 18 and Figure 19 show the performance of ML models on the Dataset Expanded (Data2) for Class 0 (OK_FRONT) and Class 1 (DEF_FRONT), respectively. Metrics include Accuracy, Precision, Sensitivity, and F1 score, demonstrating high performance with PCA = 100.

### 4.8. Comparison with Previous Work

Our results are compared with previous studies that primarily used the *Dataset Expanded*. The best models in our work using the *Dataset Original* (ResNet50 and capsule-based models with extracted parameters) achieve precision values between 97–99%, similar sensitivity, and average accuracy of 98% (Table 10, Table 11 and Table 15).

When using the *Dataset Expanded*, our ML and DL models reach even higher performance: 99.6% precision, 99.6% sensitivity, 99.6% F1-score, and 99.5% accuracy (Table 14). Compared with previous work such as Xception-CNN, DenseNet, or GoogleLeNet, which report accuracies between 97.7–99.7% on the same type of dataset, our models demonstrate equal or superior performance across all metrics.

In summary, using our approaches, we achieve highly consistent and balanced results, demonstrating the effectiveness of combining extracted features with both classical ML and DL models.

## 5. Discussion

The results of this study highlight the effectiveness of integrating statistically extracted parameters with classical ML and advanced DL approaches for the classification of surface defects in metallic cast components. Performance was evaluated on two datasets: the smaller Dataset Original and a larger, widely used dataset reported in the literature.

In the Dataset Original, applying classical ML models directly to raw images yielded moderate performance due to the high dimensionality and complexity of the data. For example, RF achieved approximately 88.85% accuracy. However, incorporating extracted and selected parameters significantly improved performance. DL models, including ResNet50 and capsule-based architectures, achieved results above 98% in accuracy and F1-score, confirming that enriching latent representations with statistical descriptors improves robustness and class discrimination.

Following the reviewer’s suggestion to clarify the experimental pipeline, we explicitly describe our procedure: the dataset was split into 80% training and 20% test sets. Within the training set, 20% was used for validation. Hyperparameters were tuned **only once** before repeating the train/test partitions. The full evaluation was then performed on the test set, and all reported results correspond to the mean and standard deviation over five repetitions. Random seeds were fixed to guarantee exact reproducibility. This approach ensures model stability, reliability, and transparency, addressing the reviewer’s concern regarding the clarity of the pipeline.

For the larger dataset, simple ML models combined with PCA (100 components) also reached very high performance without requiring additional preprocessing or feature extraction. Since this public dataset is already balanced and preprocessed with explicit train/test splits, the results are consistent and comparable with previous studies. However, fixed splits do not allow multiple repetitions to fully assess model robustness as was done with the Dataset Original.

Comparing our results with prior work, the proposed models achieve equal or superior performance in precision, recall, F1-score, and accuracy. This confirms that combining extracted parameters with ML and DL approaches is effective not only on the Dataset Original but also on datasets previously used in the literature.

Overall, these findings demonstrate that excellent performance can be achieved using different types of data: with parameter extraction for smaller, more complex datasets, and with simple ML on large preprocessed datasets. The combination of ML and DL with extracted features provides a scalable, reliable, and robust solution for automated surface inspection in metallic cast components, aligned with Industry 4.0 standards.

## 6. Conclusions

This study demonstrates that integrating statistically extracted parameters with classical ML models and advanced DL architectures substantially improves the automated classification of surface defects in metallic cast components. Using the *Dataset Original*, ML models such as RF andGB achieved high precision, accuracy, and F1-scores, showing significant improvements when combined with extracted parameters. DL models, including ResNet50 and capsule-based networks, achieved the highest performance when enriched with statistical descriptors, exceeding 98% in both accuracy and F1-score.

On larger, preprocessed datasets previously used in the literature, simple ML models combined with PCA also obtained very high performance without requiring additional feature extraction or preprocessing. While the fixed train/test splits limit repeated testing for robustness assessment, these results confirm that the proposed approaches are effective across different types of datasets.

These findings highlight the advantages of a hybrid ML/DL approach that leverages both well-engineered features and hierarchical representations learned from raw data. The approach provides a scalable, reliable, and accurate solution for non-destructive surface inspection aligned with Industry 4.0 standards. Future work will explore advanced object detection and segmentation methods, such as YOLO and Faster R-CNN, enabling not only defect classification but also precise localization and characterization, thus expanding the applicability and effectiveness of automated visual inspection systems.

## Figures and Tables

**Table 1:** Representative subset of extracted parameters used to characterize the images in multiple domains, organized by feature domain.

Parameter	Description
Statistical, Texture, and Shape	
Mean, Max, Min, Range, Std. Dev.	Basic intensity statistics
LBP, LBP Histogram	Local Binary Patterns and their histogram
GLCM (contrast, correlation, energy, homogeneity)	Gray-Level Co-occurrence Matrix features
HOG (mean)	Mean of Histogram of Oriented Gradients descriptor
Hu Moments	Scale, rotation, translation invariant moments
Edge Density	Ratio of detected edge pixels
Entropy	
Image Entropy	Measure of intensity disorder
Histogram Entropy	Entropy after various histogram transforms
FFT Entropy	Entropy of Fourier magnitude spectrum
Frequency	
Fourier Transform (mean, max, power)	Magnitude spectrum of image
Wavelet Coefficients	Multiscale decomposition coefficients
Bispectrum (mean and max values)	Second order spectral features
Other Domains: Fractal, Statistical, Complexity	
Fractal Dimension (Katz)	Measure of spatial roughness
Poincaré Features	Variability based on lagged differences
Lempel-Ziv Complexity	Algorithmic complexity of binary sequence

**Table 2:** CNN3D Architecture and Parameter Count.

Layer (Type)	Output Shape	Parameters
Conv3D	(None, 126, 126, 8, 32)	896
MaxPooling3D	(None, 63, 63, 4, 32)	0
Conv3D_1	(None, 61, 61, 2, 64)	55,360
MaxPooling3D_1	(None, 30, 30, 1, 64)	0
Flatten	(None, 57,600)	0
Dense	(None, 128)	7,372,928
Dense_1	(None, 2)	258
Total parameters		7,429,442 (28.34 MB)
Trainable parameters		7,429,442 (28.34 MB)
Non-trainable parameters		0

**Table 3:** Top 10 most important parameters according to PCA analysis.

No.	Parameter Name
1	bordes_array_0
2	FT_max_magnitud_FT
3	FT_array_0
4	Zero_Crossing
5	fft_entropy.1
6	fft_entropy
7	Hsqrt
8	lbp_hist_8
9	sbp_0
10	sbp_4

**Table 4:** Machine Learning Models and Default Hyperparameters (Independent of Target Class).

Model	Default Parameters
RF	n_estimators = 100; max_depth = None; min_samples_split = 2; min_samples_leaf = 1; max_features = auto; random_state = 42
LR	penalty = l2; C = 1.0; solver = lbfgs; max_iter = 1000; random_state = 42
K-Nearest Neighbors	n_neighbors = 5; weights = uniform; metric = minkowski; p = 2
SVM (RBF Kernel)	C = 1.0; kernel = rbf; gamma = scale; probability = True; random_state = 42
Gradient Boosting	n_estimators = 100; learning_rate = 0.1; max_depth = 3; min_samples_split = 2; min_samples_leaf = 1; subsample = 1.0; random_state = 42

**Table 5:** Results of ML models without using extracted parameters. Class 0 = Normal, Class 1 = Affected.

Model	Class	Precision (%)	Sensitivity (%)	Specificity (%)	F1 (%)	Accuracy (%)
RF	0	90.4 ± 1.1	90.4 ± 1.1	88.3 ± 0.5	90.4 ± 1.1	89.2 ± 0.6
	1	93.2 ± 0.7	88.3 ± 0.5	90.4 ± 1.1	90.7 ± 0.5	89.2 ± 0.6
LR	0	74.4 ± 0.5	74.4 ± 0.5	80.1 ± 0.0	74.4 ± 0.5	77.8 ± 0.2
	1	82.5 ± 0.3	80.1 ± 0.0	74.4 ± 0.5	81.3 ± 0.1	77.8 ± 0.2
K-Nearest Neighbors	0	87.5 ± 0.0	87.5 ± 0.0	89.1 ± 0.0	87.5 ± 0.0	88.5 ± 0.0
	1	91.4 ± 0.0	89.1 ± 0.0	87.5 ± 0.0	90.3 ± 0.0	88.5 ± 0.0
SVM (RBF Kernel)	0	82.7 ± 0.0	82.7 ± 0.0	81.4 ± 0.0	82.7 ± 0.0	81.9 ± 0.0
	1	87.6 ± 0.0	81.4 ± 0.0	82.7 ± 0.0	84.4 ± 0.0	81.9 ± 0.0
Gradient Boosting	0	86.9 ± 0.5	86.9 ± 0.5	85.1 ± 0.9	86.9 ± 0.5	85.8 ± 0.4
	1	90.7 ± 0.2	85.1 ± 0.9	86.9 ± 0.5	87.8 ± 0.4	85.8 ± 0.4

**Table 6:** Results of ML models using extracted parameters. Class 0 = Normal, Class 1 = Affected.

Model	Class	Precision (%)	Sensitivity (%)	Specificity (%)	F1 (%)	Accuracy (%)
RF	0	96.7 ± 1.6	96.7 ± 1.6	93.2 ± 2.4	96.7 ± 1.6	94.6 ± 1.4
	1	97.7 ± 1.1	93.2 ± 2.4	96.7 ± 1.6	95.4 ± 1.2	94.6 ± 1.4
LR	0	65.8 ± 5.4	65.8 ± 5.4	78.6 ± 2.9	65.8 ± 5.4	73.5 ± 3.4
	1	77.5 ± 3.1	78.6 ± 2.9	65.8 ± 5.4	78.0 ± 2.8	73.5 ± 3.4
K-Nearest Neighbors	0	66.2 ± 5.6	66.2 ± 5.6	78.3 ± 2.1	66.2 ± 5.6	73.5 ± 2.5
	1	77.7 ± 2.9	78.3 ± 2.1	66.2 ± 5.6	78.0 ± 1.9	73.5 ± 2.5
SVM (RBF Kernel)	0	67.3 ± 3.8	67.3 ± 3.8	80.5 ± 2.3	67.3 ± 3.8	75.2 ± 1.9
	1	78.7 ± 1.9	80.5 ± 2.3	67.3 ± 3.8	79.6 ± 1.6	75.2 ± 1.9
Gradient Boosting	0	97.3 ± 1.4	97.3 ± 1.4	92.9 ± 2.0	97.3 ± 1.4	94.7 ± 1.3
	1	98.1 ± 1.0	92.9 ± 2.0	97.3 ± 1.4	95.4 ± 1.2	94.7 ± 1.3

**Table 7:** Average processing time per image for different models using extracted features as input.

Method	AttnCapsNet	ConvCaps	3D Capsule Networks	Primary	Capsule 3D	Spectral
Time (s/image)	$7.10\times 10^{-4}$	$3.69\times 10^{-4}$	$3.69\times 10^{-4}$	$1.565\times 10^{-3}$	$9.17\times 10^{-4}$	$6.33\times 10^{-4}$

**Table 8:** Performance of ResNet50 models using images as input (PCA = 100). Class 0 = Normal, Class 1 = Affected.

Model	Class	Precision (%)	Sensitivity (%)	Specificity (%)	F1 (%)	Accuracy (%)
ResNet	0	64.23 ± 3.96	42.31 ± 42.31	81.41 ± 15.38	49.72 ± 32.91	65.77 ± 9.23
	1	60.00 ± 0.00	100.00 ± 0.00	0.00 ± 0.00	75.00 ± 0.00	60.00 ± 0.00

**Table 9:** Performance of Capsule-based and related Deep Learning models using raw images as input (without feature extraction). Class 0 = Normal, Class 1 = Affected.

Model	Class	Precision (%)	Sensitivity (%)	Specificity (%)	F1 (%)	Accuracy (%)
AttnCapsNet	0	90.6 ± 0.9	90.6 ± 0.9	90.4 ± 2.3	90.6 ± 0.9	90.5 ± 1.1
	1	93.5 ± 0.5	90.4 ± 2.3	90.6 ± 0.9	91.9 ± 1.0	90.5 ± 1.1
ConvCaps	0	89.8 ± 1.0	89.8 ± 1.0	90.5 ± 1.1	89.8 ± 1.0	90.2 ± 0.9
	1	93.0 ± 0.7	90.5 ± 1.1	89.8 ± 1.0	91.7 ± 0.8	90.2 ± 0.9
3D CapsNet	0	91.0 ± 1.4	91.0 ± 1.4	91.5 ± 1.2	91.0 ± 1.4	91.3 ± 1.1
	1	93.9 ± 1.0	91.5 ± 1.2	91.0 ± 1.4	92.6 ± 0.9	91.3 ± 1.1
Primary	0	89.0 ± 2.2	89.0 ± 2.2	87.8 ± 1.6	89.0 ± 2.2	88.3 ± 1.3
	1	92.3 ± 1.5	87.8 ± 1.6	89.0 ± 2.2	90.0 ± 1.2	88.3 ± 1.3
Capsule 3D	0	91.3 ± 1.2	91.3 ± 1.2	91.3 ± 0.3	91.3 ± 1.2	91.3 ± 0.3
	1	94.1 ± 0.8	91.3 ± 0.3	91.3 ± 1.2	92.7 ± 0.2	91.3 ± 0.3
Spectral	0	76.7 ± 2.5	76.7 ± 2.5	80.3 ± 3.6	76.7 ± 2.5	78.8 ± 2.9
	1	83.8 ± 2.0	80.3 ± 3.6	76.7 ± 2.5	82.0 ± 2.7	78.8 ± 2.9

**Table 10:** Performance of ResNet50 using extracted parameters. Class 0 = Normal, Class 1 = Affected.

Model	Class	Precision (%)	Sensitivity (%)	Specificity (%)	F1 (%)	Accuracy (%)
ResNet50	0	98.0 ± 2.0	98.5 ± 1.5	97.0 ± 2.0	98.2 ± 1.7	98.0 ± 1.5
	1	97.5 ± 1.8	97.0 ± 2.0	98.5 ± 1.5	97.2 ± 1.6	98.0 ± 1.5

**Table 11:** Performance of Capsule-based and related Deep Learning models using extracted features as input (with parameters). Class 0 = Normal, Class 1 = Affected.

Model	Class	Precision (%)	Sensitivity (%)	Specificity (%)	F1 (%)	Accuracy (%)
AttnCapsNet	0	99.6 ± 0.5	99.6 ± 0.5	97.3 ± 0.9	99.6 ± 0.5	98.2 ± 0.6
	1	99.7 ± 0.3	97.3 ± 0.9	99.6 ± 0.5	98.5 ± 0.5	98.2 ± 0.6
ConvCaps	0	100.0 ± 0.0	100.0 ± 0.0	97.8 ± 0.3	100.0 ± 0.0	98.7 ± 0.2
	1	100.0 ± 0.0	97.8 ± 0.3	100.0 ± 0.0	98.9 ± 0.2	98.7 ± 0.2
3D CapsNet	0	99.7 ± 0.5	99.7 ± 0.5	97.9 ± 0.3	99.7 ± 0.5	98.6 ± 0.2
	1	99.8 ± 0.3	97.9 ± 0.3	99.7 ± 0.5	98.8 ± 0.2	98.6 ± 0.2
Primary	0	100.0 ± 0.0	100.0 ± 0.0	97.0 ± 0.3	100.0 ± 0.0	98.2 ± 0.2
	1	100.0 ± 0.0	97.0 ± 0.3	100.0 ± 0.0	98.5 ± 0.1	98.2 ± 0.2
Capsule 3D	0	100.0 ± 0.0	100.0 ± 0.0	97.8 ± 0.3	100.0 ± 0.0	98.7 ± 0.2
	1	100.0 ± 0.0	97.8 ± 0.3	100.0 ± 0.0	98.9 ± 0.2	98.7 ± 0.2
Spectral	0	85.4 ± 1.1	85.4 ± 1.1	85.0 ± 1.4	85.4 ± 1.1	85.2 ± 0.7
	1	89.7 ± 0.6	85.0 ± 1.4	85.4 ± 1.1	87.3 ± 0.7	85.2 ± 0.7
Graph	0	99.6 ± 0.5	99.6 ± 0.5	97.3 ± 0.9	99.6 ± 0.5	98.2 ± 0.4
	1	99.7 ± 0.3	97.3 ± 0.9	99.6 ± 0.5	98.5 ± 0.3	98.2 ± 0.4

**Table 12:** Average processing time per image for different models using extracted features as input.

Method	AttnCapsNet	ConvCaps	3D Capsule Net.	Primary	Capsule 3D	Spectral
Time (s/image)	$7.10\times 10^{-4}$	$3.69\times 10^{-4}$	$3.69\times 10^{-4}$	$1.565\times 10^{-3}$	$9.17\times 10^{-4}$	$6.33\times 10^{-4}$

**Table 13:** Performance of CNN3D using 3D images as input. Class 0 = Normal, Class 1 = Affected.

Model	Class	Precision (%)	Sensitivity (%)	Specificity (%)	F1 (%)	Accuracy (%)
CNN3D	0	81.87 ± 2.20	91.75 ± 0.41	86.54 ± 1.92	86.51 ± 1.05	88.61 ± 1.01
	1	94.08 ± 0.20	86.54 ± 1.92	91.75 ± 0.41	90.14 ± 0.95	88.61 ± 1.01

**Table 14:** Results of ML models using extracted parameters (PCA = 100). Class 0 = Normal, Class 1 = Affected.

Model	Class	Precision (%)	Sensitivity (%)	Specificity (%)	F1 (%)	Accuracy (%)
RF	0	99.4 ± 0.2	99.4 ± 0.2	99.3 ± 0.2	99.4 ± 0.2	99.3 ± 0.2
	1	99.7 ± 0.1	99.3 ± 0.2	99.4 ± 0.2	99.5 ± 0.1	99.3 ± 0.2
K-Nearest Neighbors	0	99.2 ± 0.0	99.2 ± 0.0	98.5 ± 0.0	99.2 ± 0.0	98.7 ± 0.0
	1	99.6 ± 0.0	98.5 ± 0.0	99.2 ± 0.0	99.0 ± 0.0	98.7 ± 0.0
LR	0	83.6 ± 0.3	83.6 ± 0.3	92.7 ± 0.3	83.6 ± 0.3	89.4 ± 0.2
	1	90.7 ± 0.2	92.7 ± 0.3	83.6 ± 0.3	91.7 ± 0.2	89.4 ± 0.2
Gradient Boosting	0	96.0 ± 0.2	96.0 ± 0.2	96.2 ± 0.2	96.0 ± 0.2	96.2 ± 0.1
	1	97.6 ± 0.1	96.2 ± 0.2	96.0 ± 0.2	96.9 ± 0.1	96.2 ± 0.1
SVM	0	99.2 ± 0.0	99.2 ± 0.0	99.1 ± 0.0	99.2 ± 0.0	99.2 ± 0.0
	1	99.6 ± 0.0	99.1 ± 0.0	99.2 ± 0.0	99.3 ± 0.0	99.2 ± 0.0

**Table 15:** Comparison against previous work on the proposed dataset.

Model	Recall (%)	Precision (%)	F1 (%)	Accuracy (%)	Dataset
Proposed research (ConvCaps)	97.90	98.0	97.60	98.0	Dataset Original
Proposed research (RF)	99.60	99.60	99.60	99.50	Dataset Expanded
Xception-CNN [28]	99.62	99.62	99.62	99.72	Dataset Expanded
Voting Policy Model [29]	–	–	–	99.90	Dataset Expanded
CNN [30]	99.72	99.72	99.72	–	Dataset Expanded
ResNet [31]	98.47	97.36	97.91	98.46	Dataset Expanded
Multipath VGG19 [32]	97.72	78.01	87.37	87.39	Dataset Expanded
CNN [30]	81.71	98.12	89.14	88.52	Dataset Expanded
DenseNet [31]	99.24	99.62	99.43	99.58	Dataset Expanded
VGGNet [31]	97.71	96.24	96.97	97.76	Dataset Expanded
GoogleLeNet [31]	99.62	98.49	99.05	99.30	Dataset Expanded

## Data Availability

The original data presented in the study are openly available in Kaggle [33] at https://www.kaggle.com/datasets/ravirajsinh45/real-life-industrial-dataset-of-casting-product, accessed on 10 January 2023.
